# Randomised controlled trial of a brief theory-based online intervention to reduce self-harm

**DOI:** 10.1192/bjo.2025.2

**Published:** 2025-03-19

**Authors:** Chris Keyworth, Jessica Z. Leather, Leah Quinlivan, Rory C. O’Connor, Christopher J. Armitage

**Affiliations:** 1 School of Psychology, University of Leeds, UK; 2 NIHR Greater Manchester Patient Safety Research Collaboration, University of Manchester, Manchester Academic Health Science Centre, UK; 3 Manchester Centre for Health Psychology, School of Health Sciences, University of Manchester, UK; 4 Suicidal Behaviour Research Laboratory, Institute of Health and Wellbeing, University of Glasgow, UK; 5 Manchester University NHS Foundation Trust, Manchester Academic Health Science Centre, UK

**Keywords:** Implementation intentions, randomised controlled trial, self-harm, suicide, volitional help sheet

## Abstract

**Background:**

Forming ‘if-then’ plans has been shown to reduce self-harm among people admitted to hospital following an episode of self-harm.

**Aims:**

To explore whether the same intervention, delivered online, could prevent future self-harm among a large community sample who had previously self-harmed.

**Method:**

UK adults were recruited to a randomised controlled trial and received either an intervention to reduce self-harm or one to reduce sedentariness (control group). Randomisation was stratified to ensure both groups were representative of the UK population. There were three primary outcomes: non-suicidal self-injury (NSSI), suicidal ideation and suicide attempts, assessed at baseline and 6 months post-intervention.

**Results:**

Participants (1040) were randomised to the intervention (*n* = 520) or control (*n* = 520) group. The vast majority of people formed implementation intentions in both the experimental (*n* = 459 (88.3%)) and control (*n* = 520 (100%)) condition. Overall, the intervention did not significantly reduce the frequency of NSSI, suicidal ideation or suicide attempts. Among people who had self-harmed in the past week at follow-up, mixed analysis of covariance revealed a significant interaction between time and condition for reflective motivation, *F*(1,102) = 7.08, *P* < 0.01, *p*_n_^2^ = 0.07, such that significantly lower levels of reflective motivation were reported at follow-up in the control condition, *t*(57) = 2.42, *P* = 0.02.

**Conclusions:**

This web-based intervention has limited utility for reducing self-reported self-harm or suicidal ideation in adults with a history of self-harm. Further work is needed to improve the effectiveness of brief interventions for self-harm aimed at adults living in the community and to understand the conditions under which the intervention may or may not be effective.

Self-harm refers to a broad spectrum of behaviours and cognitions, including behaviour with suicidal intent (such as suicide attempts), behaviour without suicidal intent (e.g. non-suicidal self-injury; NSSI), and suicidal ideation.^1^ People who have previously self-harmed are also at much greater risk of future episodes of self-harm and suicide than the general population.^2^ Self-harm is therefore a serious challenge for health services in the UK.^3,4^ Although psychological therapies have been found to be effective in reducing self-harm,^5,6^ the increased demand on mental health services and soaring waiting-list times for longer-term therapies mean there is a growing need for brief interventions that can be delivered in non-clinical settings.^7^ Existing brief interventions have shown some utility in reducing suicidal ideation and behaviour but are limited by a lack of grounding in behaviour change theory and a lack of testing in non-clinical settings.^5,8^

Although there is no single reason people self-harm, there are common psychosocial triggers or critical situations (such as feelings of entrapment or defeat) that can drive episodes of self-harm.^9^ If people are provided with a strategy enabling them to respond more effectively to these critical situations, they may be less likely to engage in an act of self-harm,^10^ and there may be a subsequent reduction in the likelihood of future self-harm and suicide attempts. One means of achieving this is through the formation of implementation intentions,^11^ an approach that helps people to create an automatic response to a specified situation. Meta-analyses have demonstrated that implementation-intention-based interventions are an effective method of facilitating behaviour change across a range of behaviours,^12^ but little is known about adapting these interventions to reduce repeat self-harm. An intervention based upon implementation intentions – namely, a volitional help sheet – has been found to be effective in reducing self-harm in adults that have recently been admitted to hospital following an index episode of self-harm.^13^ Volitional help sheets provide people with a means of forming their own implementation intentions by providing critical situations they may encounter and appropriate responses to avoid self-harming. Previous trials of this intervention for self-harm have mainly been conducted with clinical patient samples and administered face-to-face in hospital discharge contexts.^13,14^ A recent study tested the effectiveness of an implementation-intentions-based intervention for community samples with a history of self-harm.^15^ Although the study found no overall differences between the experimental and control conditions in the frequency of self-harm behaviour in specified critical situations at follow-up, it highlighted the need to understand more about implementation-intention-based interventions.

According to the iceberg model of self-harm, a large proportion of self-harm episodes occur in the community;^16,17^ this creates challenges for intervention strategies, because these individuals do not seek help directly from healthcare services. However, web-based suicide prevention strategies offer a potentially cost-effective and accessible means of delivering self-harm interventions remotely to people in the community.^18^ Systematic reviews relating to self-harm and internet use suggest that online help-seeking behaviours create opportunities for intervention, although these need to be balanced with the availability of negative influences online such as contagion and triggering.^19,20^ Although internet-based interventions for self-harm have shown some promise in decreasing suicidal behaviour, a systematic review has highlighted the need for larger samples and more controlled trials.^18^ Online versions of implementation-intention-based interventions have been successfully used in previous research to change health behaviours^21^ but not in the context of self-harm in an online format. Therefore, there is a need for large-scale controlled trials of brief, web-based self-harm interventions delivered to adults in the community.

## Aims

The present research explored whether a brief theory-based intervention could help people to avoid self-harming. Although the pencil-and-paper volitional help-sheet intervention for self-harm has been shown to reduce self-harm in clinical samples, it has yet to be tested in an online format or with a community sample of adults. We also aimed to establish whether the effects of the intervention were mediated through changes in capabilities, opportunities or motivations. We hypothesised that people who formed implementation intentions would report reduced self-harm and suicidal ideation at follow-up compared with people in the control condition, and that any effects of the intervention would be mediated principally through changes in people’s capabilities, opportunities or motivations to avoid self-harming in the future.

## Method

### Design

This parallel-group (1:1) randomised controlled trial (clinicaltrials.gov identifier: NCT04420546) tested whether a volitional help sheet for self-harm was more effective in reducing suicidal ideation and behaviour compared with an active control volitional help sheet for physical activity.^22^ Participant recruitment occurred between 31 June 2020 and 1 December 2020. A survey panel company (YouGov) collected data on behalf of the research team, and all participants gave written informed consent to take part. As part of the consent process and participant briefing, participants were provided with, as part of the participant information sheet, information about a range of support services should they become worried or anxious while taking part in the research. These included 24-h helplines and support services that were open during both weekdays and weekends.

All procedures contributing to this work comply with the ethical standards of the relevant national and institutional committees on human experimentation and with the Helsinki Declaration of 1975, as revised in 2013. The study was approved by The University of Manchester research ethics committee (approval number: 2020-8446-15312). This study follows the Consolidated Standards of Reporting Trials reporting guideline for social and psychological interventions.^23^ The study formed part of a wider survey testing the acceptability of the intervention and the association of COVID-19-related fear with self-harm.^24,25^

### Participants

One thousand and forty (*n* = 1040) participants were recruited online from a pre-existing database of YouGov panel members that is representative of the UK population.^25^ Panel members were eligible to take part in the research if they were aged 18 years or older, had a self-reported history of self-harm (either in the past week, past year or longer ago), and had good verbal and written understanding of English. People who were currently residing in an in-patient facility for mental illness were excluded from taking part. A nationally representative sample from the panel were asked a screening question to ensure participants had a lifetime history of self-harm: ‘Have you ever intentionally hurt yourself/self-harmed?’. The final sample was based on respondents who answered: ‘Yes, I have’. YouGov incentivised potential participants to take part, in accordance with the company’s point system (in which respondents accumulate points for taking part in surveys, which can be exchanged for cash or entry into a prize draw). Interventions were delivered to participants online through a web-based questionnaire survey format, completed at a time and date of their choosing.

### Procedure

After participants had provided informed consent, self-reported suicidal ideation and behaviour were measured at baseline using a web-based questionnaire survey. The interventions were placed after a series of questions capturing demographic features and psychosocial measures; questions were presented in the same order to participants in both conditions. Once the questionnaire had been completed, participants in the intervention condition were presented with a volitional help sheet for self-harm, whereas participants in the active control were presented with a volitional help sheet intervention to increase physical activity. Both interventions were standardised and presented within the web-based survey. Six months after receiving the intervention, all participants were contacted and invited to take part in the follow-up survey; 778 participants accepted. The follow-up questionnaire was identical to the baseline survey, capturing demographic information, primary outcomes and secondary outcomes.

### Interventions

Participants in the intervention group were provided with a ‘volitional help sheet for self-harm’ to help them form ‘if-then plans’. The volitional help sheet for self-harm draws on theories of suicidal behaviour^26^ and theories of behaviour change^27,28^ to provide participants with a theoretically driven means of constructing implementation intentions. Previous iterations of the volitional help sheet for self-harm have used a paper-and-pencil format, where participants physically draw a line to link situations to a solution.^13^ A web-based version of the volitional help sheet for self-harm was developed with a patient and public involvement group of people with lived experience of self-harm to ensure that the intervention was understandable and acceptable.^24^ Participants in our prior work rated the intervention as positive, were confident using it, understood it and how it worked, and were confident that it would achieve its purpose.^24,25^ The present version of the volitional help sheet began with a brief statement explaining the purpose of the volitional help sheet and instructions on how to link situations that are relevant with suggested responses. As part of the statement, participants were told that identifying situations in which they were tempted to self-harm and identifying ways to overcome those temptations had been shown to help people avoid self-harming. Participants were then presented with a list of 13 common situations in which people may be tempted to self-harm (e.g. ‘If I feel the urge to self-harm when I feel defeated’). By selecting a situation, the participant was provided with a drop-down list of 13 possible solutions to choose from (e.g. ‘then I will do something else instead of self-harming’). Participants were free to make as many situation–response links as they wanted. The development of the volitional help sheet for self-harm, including the number and content of situations and solutions, has been described in full elsewhere.^24^

Participants in the active control condition were presented with a similar statement explaining the volitional help sheet but to encourage them to be more physically active. After reading the explanation and instructions, participants were presented with a list of ten situations linked to physical inactivity (e.g. ‘If I’m tempted not to be physically active when I’m under a lot of stress’) and drop-down lists of ten potential solutions (‘then I will put things around my home to remind me to be physically active’). The volitional help sheet for physical activity has been described elsewhere^22^; minor changes were made (such as the addition of drop-down menus) to adapt it for delivery online.

### Outcomes

Demographic information including sex, gender and social grade (Table 1) was obtained from participants at baseline. Three items from the British Psychiatric Morbidity Survey^29^ were used to assess the primary outcome measures of NSSI (‘Have you ever deliberately harmed yourself in any way but not with the intention of killing yourself? (i.e., self-harm) Yes/No’), suicidal ideation (‘Have you ever seriously thought of taking your life, but not actually attempted to do so? Yes/No’) and suicide attempts (‘Have you ever made an attempt to take your life, by taking an overdose of tablets or in some other way? Yes/No’). The primary outcome measures were assessed at baseline and follow-up. For each primary outcome, binary ‘yes’ and ’no’ responses were collected; those who responded ‘yes’ were also asked to indicate the timing of their most recent episode (past week, past year, longer ago) and the frequency of the episodes, in line with the British Psychiatric Morbidity Survey.

**Table 1:** Participant characteristics at baseline

Variable	Intervention, *N* (%)	Control, *N* (%)	Intervention, mean (s.d.)	Control, mean (s.d.)	Baseline differences between groups
Age, years			46.29 (14.17)	45.70 (14.26)	*t*(1038) = 0.67, *P* = 0.50, *d* = 0.04
Gender					*χ* ^2^(1) = 0.07, *P* = 0.79, odds ratio: 1.04
Male	173 (33.27)	169 (32.50)			
Female	347 (66.73)	351 (67.50)			
Social grade					*χ* ^2^(1) = 0.26, *P* = 0.61, odds ratio: 0.94
Non-manual workers	331 (63.65)	323 (62.65)			
Manual workers	189 (36.35)	197 (37.88)			
NSSI (historical)					*χ* ^2^(1) = 0.003, *P* = 0.96, odds ratio: 992
Yes	388 (74.62)	385 (74.04)			
No	127 (24.4)	125 (24.04)			
NSSI frequency			18.42 (28.91)	19.29 (29.54)	*t*(518) = −0.34, *P* = 0.73, *d* = 0.03
NSSI timing					*χ* ^2^(3) = 1.72, *P* = 0.63, *V* = 0.05
Past week	27 (5.19)	30 (5.77)			
Past year	70 (13.46)	82 (15.77)			
Longer ago	294 (56.54)	275 (52.88)			
Suicidal ideation (historical)					*χ* ^2^(1) = 0.40, *P* = 0.53, odds ratio: 1.10
Yes	394 (75.77)	386 (74.23)			
No	124 (23.85)	133 (25.58)			
Suicidal ideation frequency			11.68 (23.07)	12.25 (24.61)	*t*(547) = −0.28, *P* = 0.78, *d* = 0.02
Suicidal ideation timing					*χ* ^2^(3) = 0.86, P = 0.86, *V* = 0.03
Past week	41 (7.88)	42 (8.08)			
Past year	131 (25.19)	122 (23.46)			
Longer ago	234 (45.00)	228 (43.85)			
Suicide attempt (historical)					*χ* ^2^(1) = 0.26, *P* = 0.61, odds ratio: 1.07
Yes	215 (41.35)	205 (39.42)			
No	302 (58.08)	307 (59.04)			
Suicide attempt frequency			2.23 (4.32)	2.26 (6.60)	*t*(557) = −0.06, *P* = 0.95, *d* = 0.001
Suicide attempt timing					*χ* ^2^(3) = 2.79, P = 0.42, *V* = 0.07
Past week	5 (.96)	1 (.19)			
Past year	20 (3.85)	20 (3.85)			
Longer ago	207 (39.81)	202 (38.85)			

NSSI, non-suicidal self-harm.

The measure by Keyworth et al^30^ was used to assess people’s capabilities, opportunities and motivations with respect to reducing self-harm. In this measure, capabilities are subdivided into physical capability (e.g. skills) and psychological capability (e.g. knowledge); opportunities into physical opportunity (e.g. sufficient time) and social opportunity (e.g. support of others); and motivations into automatic motivation (e.g. habits) and reflective motivation (e.g. conscious planning). The measure comprises six items designed to explore physical capability, psychological capability, physical opportunity, social opportunity, reflective motivation and automatic motivation. The items are accompanied by a brief definition of the construct.

At follow-up, the frequencies with which critical situations were encountered were measured using a binary yes/no item; those who indicated that they had encountered a situation were asked to indicate which of the appropriate responses, if any, they used.

### Randomisation

Participants were allocated using a single sequence of random assignments^31^ to receive either the volitional help sheet intervention for self-harm or the control intervention. Web-based randomisation and enrolment were conducted by a third party (YouGov) and concealed from the research team. Double masking was implemented to blind both the research team and participants to intervention allocation.

### Analysis

Data analysis commenced after all follow-up data had been collected, and the trial was pre-registered (NCT04420546). SPSS version 25 was used for data analysis. Complete data were obtained for 778 (74.81%) participants; attrition was handled using standard intention-to-treat analysis with the last observation carried forward.

The research team conducted randomisation checks (chi-squared tests for categorical variables and *t*-tests for continuous variables) on all outcome and demographic variables to determine whether any baseline differences occurred between the two conditions. Frequency counts were examined to identify which critical situations were encountered and which appropriate responses were used. Missing data were imputed using last observation carried forward.

The primary outcomes of the study were continuous frequency measures of self-harm (non-suicidal self-harm, suicidal ideation and suicide attempts). The secondary outcome measures were assessments of people’s capabilities, opportunities and motivations to avoid self-harming in the future. All primary and secondary outcomes were tested using mixed analyses of variance (ANCOVAs), with condition (intervention versus control) as the between-participants variable, and time (baseline versus 6-month follow-up) as the within-participants variable. For each of these analyses, social grade, age and gender were used as covariates.

The effects of the intervention on implementation intention formation were tested using multivariate analysis of covariance (MANCOVA) with condition (intervention versus control) as the between-participants variable, and participant-reported use of each of the 13 appropriate solutions as the dependent variable. Social grade, age, gender and self-harm at baseline (any measure) were used as covariates.

## Results

**Fig. 1:**
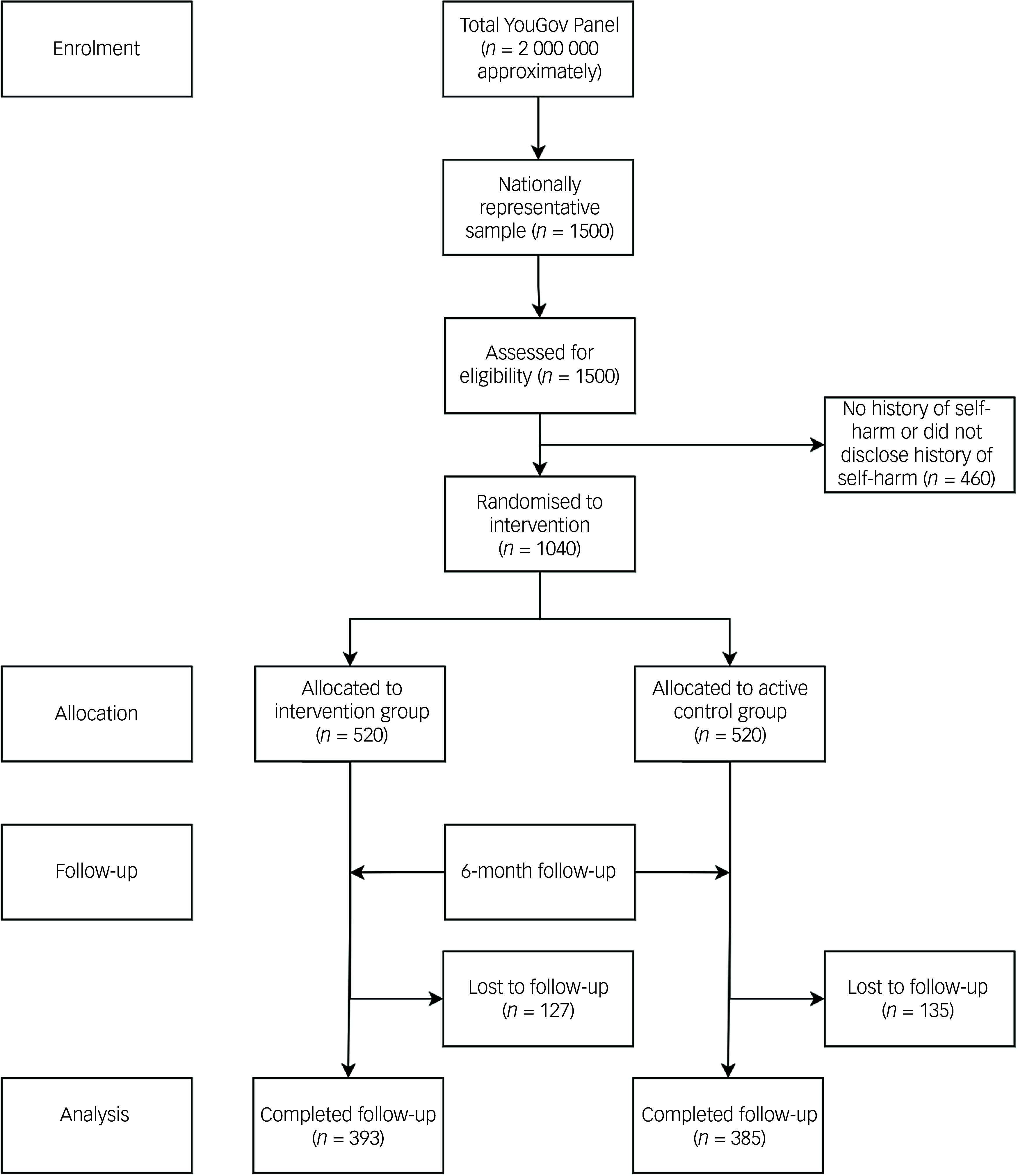
Participant flow diagram.

Of the 1040 people deemed eligible to participate, 520 were randomly assigned to the intervention group and 520 to the control group in June 2020 (Fig. 1). Follow-up data collection occurred during December 2020, marking the end of the trial. Follow-up data were collected from 393 (75.58%) participants in the intervention group and 385 (74.00%) in the control group. There were no significant differences between the participants who dropped out of the study and those that remained in the study on any outcome measure or demographic feature (all *P* > 0.05). There were no unintended effects or adverse events recorded by the research team in either the intervention or the control group.

### Baseline data

Table 1 displays the sample characteristics at baseline. All participants reported a lifetime history of self-harm on at least one of the self-report measures. Of the total sample, 74.33% (*n* = 773) reported a lifetime history of NSSI, 74.71% (*n* = 780) reported a lifetime history of suicidal ideation and 40.38% (*n* = 420) reported a lifetime history of a suicide attempt. Full demographic features are detailed in Supplementary File 1 available at https://doi.org/10.1192/bjo.2025.2. There were no significant differences between the experimental groups on any outcome measure or demographic feature at baseline (Table 1), indicating successful randomisation. The vast majority of people formed implementation intentions in both the experimental condition (*n* = 459 (88.3%)) and the control condition (*n* = 520 (100%)) and therefore completed the intervention.

### Main outcomes

Self-harm related outcomes at 6 months follow-up according to group are summarised in Table 2.

**Table 2:** Comparison between baseline and follow-up measures for non-suicidal self-injury (NSSI), suicidal ideation and suicide attempts between the intervention group and control group^a^

	Baseline		Follow-up	
Category	Intervention	Control	Intervention	Control
NSSI				
Yes	388 (74.62)	385 (74.04)	300 (77.32)	292 (76.84)
No	127 (24.4)	125 (24.04)	88 (22.68)	88 (23.16)
NSSI frequency, mean (s.d.)	18.42 (28.91)	19.29 (29.54)	19.81 (28.56)	21.89 (32.73)
NSSI timing				
Past week	27 (5.19)	30 (5.77)	15 (4.81)	26 (8.41)
Past year	70 (13.46)	82 (15.77)	53 (16.99)	67 (21.68)
Longer ago	294 (56.54)	275 (52.88)	230 (73.72)	203 (65.70)
Suicidal ideation				
Yes	394 (75.77)	386 (74.23)	301 (76.79)	295 (76.62)
No	124 (23.85)	133 (25.58)	91 (23.21)	90 (23.38)
Ideation frequency, mean (s.d.)	11.68 (23.07)	12.25 (24.61)	10.27 (21.82)	11.31 (23.42)
Ideation timing				
Past week	41 (7.88)	42 (8.08)	28 (8.72)	31 (9.90)
Past year	131 (25.19)	122 (23.46)	93 (28.97)	93 (29.71)
Longer ago	234 (45.00)	228 (43.85)	188 (58.57)	176 (56.23)
Suicide attempt				
Yes	215 (41.35)	205 (39.42)	167 (43.04)	151 (39.95)
No	302 (58.08)	307 (59.04)	221 (56.96)	227 (60.05)
Attempt frequency, mean (s.d.)	2.23 (4.32)	2.26 (6.60)	2.31 (7.59)	1.68 (3.19)
Attempt timing				
Past week	5 (.96)	1 (.19)	2 (.97)	1 (.52)
Past year	20 (3.85)	20 (3.85)	13 (6.28)	12 (6.25)
Longer ago	207 (39.81)	202 (38.85)	164 (79.23)	149 (77.60)

a. All data are presented at *n* (%) unless otherwise stated. Physical activity data were also collected at follow-up, but no differences were observed between the intervention and control condition; *t*(402) = 0.41, *P* = 0.68, *d* = 0.04.

#### Frequency of NSSI, suicidal ideation and suicide attempts

Mixed ANCOVA found no significant interaction between time and condition for the frequency of NSSI (*F*(1,773) = 0.87, *P* = 0.35, *P*_n_^2^ = 0.001). No significant main effects of time (*F*(1,773) = 0.62, *P* = 0.43, *P*_n_^2^ = 0.001) or condition (*F*(1,773) = 0.22, *P* = 0.64, *P*_n_^2^ < 0.001) on the frequency of NSSI were found. For frequency of suicidal ideation, mixed ANCOVA found no significant interaction between time and condition (*F*(1,776) = 0.26, *P* = 0.61, *P*_n_^2^ < 0.001). No significant main effects of time (*F*(1,776) = 0.59, *P* = 0.42, *P*_n_^2^ = 0.001) or condition (*F*(1,776) = 0.22, *P* = 0.64, *P*_n_^2^ < 0.001) on the frequency of suicidal ideation were found. Furthermore, mixed ANCOVA found no significant interaction between time and condition for the frequency of suicide attempts (*F*(1,419) =2.79, *P* = 0.09, *P*_n_^2^ = 0.007). No significant main effects of time (*F*(1,419) =1.08, *P* = 0.30, *P*_n_^2^ = 0.003) or condition (*F*(1,419) = 0.16, *P* = 0.69, *P*_n_^2^ < 0.001) on the frequency of suicide attempts were found.

Follow-up subgroup mixed ANCOVAs were conducted among people who had self-harmed in the past week at follow-up (on any measure), to examine any significant interactions between time and condition with respect to the frequency of NSSI, frequency of suicidal ideation and frequency of suicidal attempts. In addition, we re-ran the analyses excluding participants who did not form an implementation intention at baseline (*n* = 61). All ANCOVAs were non-significant.

#### Capabilities, opportunities and motivations

For physical capability, mixed ANCOVA found no significant interaction between time and condition (*F*(1,927) = 0.16, *P* = 0.69, *P*_n_^2^ < 0.001). No significant main effects of time (*F*(1,927) = 0.47, *P* = 0.50, *P*_n_^2^ < 0.01) or condition (*F*(1,927) = 0.96, *P* = 0.33, *P*_n_^2^ < 0.01) on physical capability scores were found. For psychological capability, mixed ANCOVA found no significant interaction between time and condition (*F*(1,941) = 0.45, *P* = 0.50, *P*_n_^2^ < 0.001). No significant main effects of time (*F*(1,941) = 0.58, *P* = 0.47, *P*_n_^2^ < 0.01) or condition (*F*(1,941) = 2.08, *P* = 0.15, *P*_n_^2^ < 0.01) on physical capability scores were found. For physical opportunity, mixed ANCOVA found no significant interaction between time and condition (*F*(1,885) = 0.96, *P* = 0.33, *P*_n_^2^ < 0.01). No significant main effects of time (*F*(1,885) = 0.1.18, *P* = 0.28, *P*_n_^2^ < 0.01) or condition (*F*(1,885) = 0.64, *P* = 0.42, *P*_n_^2^ < 0.001) on physical opportunity scores were found. For social opportunity, mixed ANCOVA found no significant interaction between time and condition (*F*(1,884) = 0.09, *P* = 0.76, *P*_n_^2^ < 0.001). No significant main effects of time (*F*(1,884) = 0.01, *P* = 0.92, *P*_n_^2^ < 0.001) or condition (*F*(1,884) = 0.028, *P* = 0.60, *P*_n_^2^ < 0.001) on physical opportunity scores were found. For reflective motivation, mixed ANCOVA found no significant interaction between time and condition (*F*(1,917) = 0.02, *P* = 0.89, *P*_n_^2^ < 0.001). No significant main effects of time (*F*(1,917) = 0.79, *P* = 0.38, *P*_n_^2^ < 0.001) or condition (*F*(1,917) = 2.03, *P* = 0.16, *P*_n_^2^ < 0.01) on reflective motivation scores were found. For automatic motivation, mixed ANCOVA found no significant interaction between time and condition (*F*(1,915) = 0.85, *P* = 0.36, *P*_n_^2^ < 0.01). No significant main effects of time (*F*(1,915) = 0.37, *P* = 0.54, *P*_n_^2^ < 0.001) or condition (*F*(1,915) = 1.88, *P* = 0.17, *P*_n_^2^ < 0.01) on automatic motivation scores were found.

Follow-up subgroup mixed ANCOVAs were conducted among people who had self-harmed in the past week at follow-up (on any measure), to examine any significant interactions between time and condition with respect to measures of capabilities, opportunities and motivations. All ANCOVAs, with the exception of that for reflective motivation, were non-significant. For reflective motivation, mixed ANCOVA revealed a significant interaction between time and condition, *F*(1,102) = 7.08, *P* < 0.01, *P*_n_^2^ = 0.07 (Table 3). Further *t*-tests to assess differences at follow-up between groups showed significant differences between baseline and follow-up scores across groups, with lower levels of reflective motivation reported at follow-up in the control condition (baseline: mean 6.17, s.d. 2.77; follow-up: mean 5.17, s.d. 3.01, *t*(57) = 2.42, *P* = 0.02). No significant differences were observed in the intervention condition (baseline: mean 5.65, s.d. 3.36; follow-up: mean 6.04, s.d. 2.90, *t*(48) = −1.22, *P* = 0.23).

**Table 3:** Comparison between baseline and follow-up measures of self-reported capability, opportunity and motivation to reduce self-harm between the intervention group and control group^a^

	Full sample				Subgroup analysis^b^			
	Baseline		Follow-up		Baseline		Follow-up	
Category	Intervention	Control	Intervention	Control	Intervention	Control	Intervention	Control
Physical opportunity	7.29 (2.81)	7.10 (2.87)	7.21 (2.92)	7.22 (2.90)	6.45 (3.10)	6.61 (3.22)	5.98 (3.09)	6.09 (3.33)
Social opportunity	6.48 (3.21)	6.37 (3.14)	6.42 (3.30)	6.41 (3.09)	5.08 (3.17)	5.88 (2.95)	4.64 (3.15)	5.39 (3.34)
Reflective motivation	7.41 (2.96)	7.20 (2.87)	7.48 (2.92)	7.29 (2.95)	5.65 (3.36)	6.17 (2.77)	6.04 (2.90)	5.17 (3.01)
Automatic motivation	6.92 (3.13)	6.58 (3.25)	6.92 (3.22)	6.72 (3.19)	5.30 (3.34)	5.36 (3.26)	4.78 (3.38)	4.58 (3.25)
Physical capability	7.98 (2.47)	7.86 (2.41)	8.12 (2.39)	7.91 (2.43)	6.79 (2.89)	6.43 (2.79)	6.50 (2.85)	6.36 (2.82)
Psychological capability	7.39 (2.61)	7.18 (2.62)	7.54 (2.57)	7.26 (2.69)	5.94 (2.93)	5.54 (2.85)	5.88 (2.87)	4.97 (2.94)

a.All data are presented as mean (s.d.) For the subgroup analysis, the condition × time interaction associated with reflective motivation was significant, *F*(1,102) = 7.08, *P* < 0.01, *P*_n_^2^ = 0.07.

b.People who had self-harmed in the past week (on any measure).

We also re-ran these analyses excluding participants who did not form an implementation intention at baseline (*n* = 61). Mirroring the subgroup analyses, for reflective motivation, the interaction between time and condition remained significant: *F*(1,100) = 8.05, *P* < 0.01, *P*_n_^2^ = 0.07. Further *t*-tests to assess differences at follow-up between groups showed significant differences between baseline and follow-up scores across groups, with lower levels of reflective motivation reported at follow-up in the control condition (baseline: mean 6.09, s.d. 2.77; follow-up: mean 5.22, s.d. 3.00, *t*(57) = 2.42, *P* < 0.01). No significant differences were observed in the intervention condition (baseline: mean 5.68, s.d. 3.26; follow-up: mean 6.21, s.d. 2.87, *t*(46) = −1.63, *P* = 0.06). In addition, for psychological capability, mixed ANCOVA revealed a significant interaction between time and condition: *F*(1,100) = 4.12, *P* < 0.05, *P*_n_^2^ = 0.04. Further *t*-tests to assess differences at follow-up between groups showed significant differences between baseline and follow-up scores across groups, with lower levels of psychological capability were reported at follow-up in the control condition (baseline: mean 5.59, s.d. 2.85; follow-up: mean 5.02, s.d. 2.94, *t*(57) = 2.11, *P* = 0.02). No significant differences were observed in the intervention condition (baseline: mean 5.89, s.d. 2.77; follow-up: mean 6.17, s.d. 2.67, *t*(46) = −0.93, *P* = 0.18).

#### Effects of the intervention on implementation intention formation

The effects of the intervention on implementation intention formation were tested using MANCOVA with condition (intervention versus control) as the between-participants variable and participant-reported use of each of the 13 appropriate solutions as the dependent variable. Using Pillai’s trace, MANCOVA showed there were no significant main effects for the manipulation on any dependent variable (*V* = 0.010, *F*(13,1022) = 0.770, *P* = 0.69, *P*_n_^2^ = 0.01).

Follow-up subgroup MANCOVAs were conducted among people who had self-harmed in the past week at follow-up (on any measure), to examine the effects of the intervention on implementation intention formation. Using Pillai’s trace, MANCOVA indicated that there were significant differences between the intervention and control group with respect to critical situations encountered (*V* = 0.205, *F*(13,95) = 1.88, *P* < 0.05, *P*_n_^2^= 0.21). Univariate *F* tests revealed significant differences between groups (experimental versus control) in the use of implementation intentions, with participants in the experimental group reporting significantly higher use of solution 5 (mean 0.33, s.d. 0.82; mean 0.09, s.d. 0.35), solution 9 (mean 0.45, s.d. 1.14; mean 0.08, s.d. 0.33) and solution 10 (mean 0.47, s.d. 1.10; mean 0.11, s.d. 0.48). Although a large number of people did not form implementation intentions, which affected the overall means, nevertheless, people reported more use of these solutions in the experimental compared with the control group.

## Discussion

This randomised controlled trial investigated the efficacy of a web-based volitional help sheet to reduce self-harm in a community sample of adults with a history of self-harm. The principal finding was that the web-based volitional help sheet for self-harm did not reduce self-harm related outcomes compared with a web-based volitional help sheet for physical activity.

To date, research testing the effectiveness of the volitional help sheet for self-harm has predominantly been conducted only with clinical samples at discharge from hospital following an episode of self-harm.^13,14^ A more recent study using a community sample with a history of self-harm^15^ found no overall differences between the experimental and control conditions with respect to the frequency of self-harm behaviour but highlighted the need to understand more about implementation-intention-based interventions. The present study extends the evidence base to provide additional support for using nationally representative community samples of adults; this is important because there is an urgent need for interventions that are effective at reducing self-harm that occurs outside the reach of healthcare services.^17,32^ This study also demonstrates the need to understand the conditions under which the intervention may or may not be effective.

Our findings are consistent with previous studies that found no overall effect of the volitional help sheet for self-harm.^14,15^ However, our finding that NSSI, suicidal ideation and suicide attempts did not significantly increase following the intervention provides reassurance that the potential harms of the intervention are low in a community sample. This is in contrast to findings from a clinical sample, which suggested that a more tailored approach may be helpful, with the volitional help sheet being particularly helpful for people admitted to hospital with self-harm in the prior 10 years.^14^ It was further encouraging that no significant increases in self-harm outcomes were observed; this was in contrast to the increasing levels of psychological distress reported during the early phases of the COVID-19 pandemic,^33^ a time during which data collection for the present study occurred.

An important finding is that the control condition reported significantly lower levels of reflective motivation to avoid self-harming at follow-up. Although the intervention group reported higher levels of reflective motivation to avoid self-harming in the future, this difference was not statistically significant. The capabilities, opportunities and motivations model of behaviour change describes reflective motivation as a conscious influence on behaviour that captures how much people want, intend and plan to do something^34^ and consequently may be an important mechanism for future interventions. Research is required to further understand the mediators of implementation-intention-based interventions.

Although the web-based volitional help sheet used in this study provided participants with stable cues on which to base their if-then plans, the current format of the intervention did not utilise other behaviour change techniques to support motivation^35^; techniques such as reminders (follow-up prompts) are potential means of supporting the accessibility of implementation intentions delivered through digital interventions.^36^ The addition of such features in future research could also be combined with further improvements to correct formatting issues such as situation–response customisation and instruction coherence that were highlighted by trial participants.^24^

### Limitations

Although the study had several strengths, it also had potential limitations. First, the outcome measures for self-harm did not necessarily capture changes in self-harm behaviour accurately over the 6 -month study period; the timing measures were adapted from the British Psychiatric Morbidity Survey, which measures self-harm ‘in the last week’, or ‘in the last year’. Items that can detect self-harm more sensitively, such as the Inventory of Statements about Self-Injury,^37^ could provide a more detailed illustration of self-harm behaviour during the period between baseline and follow-up. Second, although participants were sampled to be representative of people who self-harm in the UK, the generalisability of the findings is uncertain^38^; the majority (*n* = 569, 55.74%) of the sample had not self-harmed or experienced suicidal ideation (*n* = 462, 44.43%) within the past year at baseline; the intervention may have had limited utility for these participants. In addition, a number of participants self-reported long-term health conditions such as depression (*n* = 432; 41.5%) and anxiety (*n* = 400; 38.5%; data presented in Supplementary File 1); this may suggest a need to explore the utility of more targeted interventions for people reporting long-term health conditions, which may affect how they engage with the intervention.

Future research should aim to trial the intervention among subgroups in which the need for coping mechanisms is greatest, such as those who have self-harmed more recently.^39^ In addition, it may be useful to trial the intervention in settings such as primary care, where there may be opportunities for early identification of self-harm and appropriate interventions but a current lack of the necessary support and resources.^40^ However, this needs to be balanced with functional improvements to the intervention: first, to address the increased perceived burden of the intervention for those who have self-harmed in the past year;^24^ and, second, to explore ways of improving accessibility to the intervention, such as making it more accessible offline (not requiring internet access)^32^ and exploring the role of digital literacy in engaging with the intervention.

In conclusion, this study has demonstrated that a web-based volitional help sheet for self-harm has limited utility for adults in the community that have previously self-harmed. More research is required to improve the capacity of the volitional help sheet to support people’s capabilities, opportunities and motivations to avoid self-harm. Future work should aim to refine the delivery of the intervention in ways that are acceptable and effective for people living in the community.

## Supporting information

Keyworth et al. supplementary material Keyworth et al. supplementary material

## Data Availability

The data-sets used and/or analysed during the current study are available from the corresponding author on reasonable request.
