# Supplementary material for: Randomised controlled trial of a brief theory-based online intervention to reduce self-harm
Source: BJPsych Open. 2025 Mar 19;11(2):e63. doi: 10.1192/bjo.2025.2 (PMC12001932; doi:10.1192/bjo.2025.2)
Supplement: Keyworth et al. supplementary material [file S205647242500002Xsup001.docx]

**Supplement information (1)**

Table 1: Full demographic information

| **Category** | **Intervention**  ***N* (%)** | **Control**  ***N* (%)** | **Intervention M (SD)** | **Control M (SD)** |
| --- | --- | --- | --- | --- |
| **Age** |  |  |  |  |
| 18-24 | 31 (5.96) | 26 (5.00) |  |  |
| 25-34 | 110 (21.15) | 119 (22.88) |  |  |
| 35-44 | 111 (21.35) | 120 (23.08) |  |  |
| 45-54 | 94 (18.08) | 104 (20.00) |  |  |
| 55+ | 174 (33.46) | 151 (29.04) |  |  |
| Average |  |  | 46.29 (14.17) | 45.70 (14.26) |
| **Gender** |  |  |  |  |
| Male | 173 (33.27) | 169 (32.50) |  |  |
| Female | 347 (66.73) | 351 (67.50) |  |  |
| **Ethnicity** |  |  |  |  |
| White British | 440 (84.62) | 438 (84.23) |  |  |
| Any other white background | 37 (7.12) | 26 (5.00) |  |  |
| White and Black Caribbean | 0 (.00) | 2 (.38) |  |  |
| White and Asian | 3 (.58) | 6 (1.15) |  |  |
| Any other mixed background | 5 (.96) | 1 (.19) |  |  |
| Indian | 0 (.00) | 1 (.19) |  |  |
| Pakistani | 0 (.00) | 7 (1.35) |  |  |
| Bangladeshi | 0 (.00) | 1 (.19) |  |  |
| Black African | 1 (.19) | 0 (.00) |  |  |
| Any other black background | 1 (.19) | 0 (.00) |  |  |
| Chinese | 2 (.38) | 0 (.00) |  |  |
| Other ethnic group | 4 (.77) | 2 (.38) |  |  |
| Prefer not to say | 3 (.58) | 4 (.77) |  |  |
| Missing | 24 (4.62) | 32 (6.15) |  |  |
| **Sexuality** |  |  |  |  |
| Heterosexual | 396 (76.15) | 414 (79.62) |  |  |
| Gay or lesbian | 36 (6.92) | 32 (6.15) |  |  |
| Bisexual | 49 (9.42) | 42 (8.08) |  |  |
| Other | 18 (3.46) | 17 (3.27) |  |  |
| Prefer not to say | 21 (4.04) | 15 (2.88) |  |  |
| **Social Grade** |  |  |  |  |
| A | 71 (13.65) | 80 (15.38) |  |  |
| B | 131 (25.19) | 102 (19.62) |  |  |
| C1 | 129 (24.81) | 141 (27.12) |  |  |
| C2 | 67 (12.88) | 65 (12.50) |  |  |
| D | 39 (7.50) | 39 (7.50) |  |  |
| E | 83 (15.96) | 93 (17.88) |  |  |
| **Long-term condition** |  |  |  |  |
| Arthritis (rheumatoid and osteoarthritis) | 90 (17.31) | 95 (18.27) |  |  |
| Osteoporosis | 13 (2.50) | 9 (1.73) |  |  |
| Asthma | 83 (15.96) | 78 (15.00) |  |  |
| Respiratory disease (COPD, ARDS, emphysema) | 9 (1.73) | 13 (2.50) |  |  |
| Angina | 3 (.58) | 9 (1.73) |  |  |
| Congestive heart failure/ disease | 7 (1.35) | 7 (1.35) |  |  |
| Heart attack (myocardial infarct) | 2 (.38) | 7 (1.35) |  |  |
| Neurological disease | 12 (2.31) | 14 (2.69) |  |  |
| Stroke or TIA | 10 (1.92) | 8 (1.54) |  |  |
| Peripheral vascular disease | 3 (.58) | 2 (.38) |  |  |
| Diabetes types I and II | 47 (9.04) | 41 (7.88) |  |  |
| Upper gastrointestinal disease | 31 (5.96) | 39 (7.50) |  |  |
| Depression | 212 (40.77) | 220 (42.31) |  |  |
| Anxiety or panic disorders | 191 (36.73) | 209 (40.19) |  |  |
| Visual impairment | 19 (3.65) | 20 (3.85) |  |  |
| Hearing impairment | 21 (4.04) | 15 (2.88) |  |  |
| Degenerative disc disease | 42 (8.08) | 38 (7.31) |  |  |
| Obesity and/or body mass index >30 | 90 (17.31) | 116 (22.31) |  |  |
| None of the above | 146 (28.08) | 135 (25.96) |  |  |
| Prefer not to say | 4 (.77) | 3 (.58) |  |  |
| **Region** |  |  |  |  |
| North East | 18 (3.46) | 12 (2.31) |  |  |
| North West | 36 (6.92) | 53 (10.19) |  |  |
| Yorkshire and Humber | 65 (12.50) | 47 (9.04) |  |  |
| East Midlands | 40 (7.69) | 32 (6.15) |  |  |
| West Midlands | 41 (7.88) | 34 (6.54) |  |  |
| East of England | 47 (9.04) | 43 (8.27) |  |  |
| London | 52 (10.00) | 59 (11.35) |  |  |
| South East | 85 (16.35) | 77 (14.81) |  |  |
| South West | 65 (12.50) | 64 (12.31) |  |  |
| Wales | 28 (5.38) | 44 (8.46) |  |  |
| Scotland | 32 (6.15) | 43 (8.27) |  |  |
| Northern Ireland | 11 (2.12) | 12 (2.31) |  |  |
| **Education** |  |  |  |  |
| No formal qualifications | 17 (3.27) | 21 (4.04) |  |  |
| Youth training certificate/skillseekers | 3 (.58) | 4 (.77) |  |  |
| Recognised trade apprenticeship completed | 3 (.58) | 6 (1.15) |  |  |
| Clerical and commercial | 4 (.77) | 3 (.58) |  |  |
| City & Guilds certificate | 15 (2.88) | 16 (3.08) |  |  |
| City & Guilds certificate - advanced | 6 (1.15) | 6 (1.15) |  |  |
| ONC | 4 (.77) | 0 (.00) |  |  |
| CSE grades 2-5 | 6 (1.15) | 8 (1.54) |  |  |
| CSE grade 1, GCE O level, GCSE, School Certificate | 56 (10.77) | 52 (10.00) |  |  |
| Scottish Ordinary/ Lower Certificate | 1 (.19) | 3 (.58) |  |  |
| GCE A level or Higher Certificate | 53 (10.19) | 69 (13.27) |  |  |
| Scottish Higher Certificate | 5 (.96) | 5 (.96) |  |  |
| Nursing qualification (e.g. SEN, SRN, SCM, RGN) | 10 (1.92) | 10 (1.92) |  |  |
| Teaching qualification (not degree) | 10 (1.92) | 4 (.77) |  |  |
| University diploma | 32 (6.15) | 23 (4.42) |  |  |
| University or CNAA first degree (e.g. BA, B.Sc, B.Ed) | 154 (29.62) | 142 (27.31) |  |  |
| University or CNAA higher degree (e.g. M.Sc, Ph.D) | 90 (17.31) | 86 (16.54) |  |  |
| Other technical, professional or higher qualification | 42 (8.08) | 49 (9.42) |  |  |
| Don't know | 4 (.77) | 4 (.77) |  |  |
| Prefer not to say | 5 (.96) | 9 (1.73) |  |  |
